# Systematic Profiling of Immune Risk Model to Predict Survival and Immunotherapy Response in Head and Neck Squamous Cell Carcinoma

**DOI:** 10.3389/fgene.2020.576566

**Published:** 2020-10-16

**Authors:** Xingyu Liu, Jiarui Chen, Wei Lu, Zihang Zeng, Jiali Li, Xueping Jiang, Yanping Gao, Yan Gong, Qiuji Wu, Conghua Xie

**Affiliations:** ^1^Department of Radiation and Medical Oncology, Zhongnan Hospital of Wuhan University, Wuhan, China; ^2^Department of Gastrointestinal Surgery II, Renmin Hospital of Wuhan University, Wuhan, China; ^3^Department of Biological Repositories, Zhongnan Hospital of Wuhan University, Wuhan, China; ^4^Hubei Key Laboratory of Tumor Biological Behaviors, Zhongnan Hospital of Wuhan University, Wuhan, China; ^5^Hubei Cancer Clinical Study Center, Zhongnan Hospital of Wuhan University, Wuhan, China

**Keywords:** risk model, head and neck squamous cell carcinoma, human papillomavirus, prognosis, immunotherapy response

## Abstract

**Background and Purpose:**

Head and neck squamous carcinoma (HNSCC), characterized by immunosuppression, is a group of highly heterogeneous cancers. Although immunotherapy exerts a promising influence on HNSCC, the response rate remains low and varies in assorted primary sites. Immunological mechanisms underlying HNSCC pathogenesis and treatment response are not fully understood. This study aimed to develop a differentially expressed genes (DEGs)–based risk model to predict immunotherapy efficacy and stratify prognosis of HNSCC patients.

**Materials and Methods:**

The expression profiles of HNSCC patients were downloaded from The Cancer Genome Atlas (TCGA) database. The tumor microenvironment and immune response were estimated by cell type identification via estimating relative subset of known RNA transcripts (CIBERSORT) and immunophenoscore (IPS). The differential expression pattern based on human papillomavirus status was identified. A DEGs-based prognostic risk model was developed and validated. All statistical analyses were performed with R software (version 3.6.3).

**Results:**

By using the TCGA database, we identified *DKK1*, *HBEGF*, *RNASE7*, *TNFRSF12A*, *INHBA*, and *IPIK3R3* as DEGs that were associated with patients’ overall survival (OS). Patients were stratified into the high- and low-risk subgroups according to a DEGs-based prognostic risk model. Significant difference in OS was found between the high- and low-risk patients (1.64 vs. 2.18 years, *P* = 0.0017). In multivariate Cox analysis, the risk model was an independent prognostic factor for OS (hazard radio = 1.06, 95% confidence interval [1.02–1.10], *P* = 0.004). More CD8^+^ T cells and regulatory T cells were observed in the low-risk group and associated with a favorable prognosis. The IPS analysis suggested that the low-risk patients possessed a higher IPS score and a higher immunoreactivity phenotype, which were correlated with better immunotherapy response.

**Conclusion:**

Collectively, we established a reliable DEGs-based risk model with potential prognostic value and capacity to predict the immunophenotype of HNSCC patients.

## Introduction

Squamous cell carcinomas, originating from the oral cavity, oropharynx, larynx, or hypopharynx, are collectively referred to as head and neck squamous cell carcinoma (HNSCC). HNSCC has become the sixth leading cancer by incidence worldwide, whereas only fewer than 50% of patients could survive for 5 years (Kamangar et al., 2006). About two-thirds of HNSCC patients are diagnosed at late stages with poor prognosis. Patients with locally advanced stage HNSCC are treated with surgery and postoperative radiotherapy. Targeted drugs, such as cetuximab, an epidermal growth factor receptor (EGFR)–specific antibody, have been utilized for HNSCC and reached a limited response rate, possibly due to its clinical heterogeneity (Licitra et al., 2011). Novel therapeutic strategies are in urgent need.

Recent studies documented that the programmed death 1 (PD-1) inhibitors such as pembrolizumab and nivolumab significantly improve the overall survival (OS) of recurrent/metastatic HNSCC patients. However, PD-1 inhibitors have merely low to moderate response rates (Chow et al., 2016; Seiwert et al., 2016). The factors that determine treatment response to PD-1 inhibitors in HNSCC are not clear. The tumor microenvironment (TME) of HNSCC is highly heterogeneous and predominantly immunosuppressive, characterized by macrophages and myeloid-derived suppressor cell recruitment (Canning et al., 2019), T cell and natural killer (NK) cell dysfunction, regulatory T cell (Treg) activation (Reichert et al., 2002), and alteration in cytokine release such as enhanced interleukin-10 (IL-10) and IL-6 production and reduced transforming growth factor β (TGF-β) and IL-12 secretion (Lathers and Young, 2004; Varilla et al., 2013). These changes in TME might lead to a limited PD-1 inhibitor treatment response. On the other hand, high-level expression of PD-1 and/or cytotoxic T-lymphocyte antigen 4 (CTLA4) on T cells, as well as programmed death ligand 1 (PD-L1) up-regulation on malignant cells and immune cells in a portion of HNSCC patients, has been reported, which might dictate the treatment efficacy of immune checkpoint inhibitors (ICIs) (Badoual et al., 2013; Concha-Benavente et al., 2016; Mandal et al., 2016). There is still a lack of a useful risk model that could predict the response to immunotherapy and provide prognostic information on HNSCC patients. Thus, deeper understanding of genomic alterations and immune-related markers might help develop such risk models.

Increased human papillomavirus (HPV) infection together with tobacco and alcohol abuse has been identified as the most important risk factors. HPV infection is mostly associated with oropharyngeal cancer (Tanaka and Alawi, 2018). A recent study in France showed 43.1% HPV-positive rate in HNSCC patients. Compared with HPV-negative HNSCC patients, HPV-positive HNSCC patients have a better OS and disease-free survival (Mirghani et al., 2019). HPV viral genome could integrate into the host genome and alter gene expression, affecting HNSCC TME. For example, HPV infection significantly impairs IL-6 and macrophage colony-stimulating factor release, creating an immunosuppressive microenvironment (Smola, 2017). Besides, HPV-positive HNSCC has high immune infiltrates such as Tregs and CD56^+^ NK cells (Mandal et al., 2016). Unfortunately, the mechanism underlying HPV–TME interaction is still unclear. Our study aims to construct a new prognostic risk model in HNSCC. The respective relations between risk model, clinical characteristics, and OS were investigated based on HPV-related genomic expression alteration. Cell type identification by estimating relative subset of known RNA transcripts (CIBERSORT) was applied to quantify the immune cell infiltration status in HNSCC TME. Finally, we explored the hub immune biomarkers to have an in-depth understanding of HNSCC immunotherapy.

## Materials and Methods

### Patients Data

The gene expression data and clinical information of 479 HNSCC patients were downloaded from The Cancer Genome Atlas (TCGA) data portal^1^. We further obtained 100 patients with known HPV status determined by p16 IHC staining and 83 patients with known HPV status determined by *in situ* hybridization (ISH). The differences between two methods were compared, and p16 testing was chosen for further analysis. The list of immune-related genes (IRGs) was achieved from the Immunology Database and Analysis Portal (ImmPort) database that provides more than 2,000 IRGs and annotations^2^.

### Differential Gene Analysis and Functional Enrichment of DEGs

The differentially expressed genes (DEGs) in HPV-positive and HPV-negative HNSCC tissues were calculated using the limma package of R software^3^. mRNAs with an adjusted *P* < 0.05 and | log2 (fold change)| >1 were figured out as DEGs. Immune-related DEGs were identified by venn package. Heat maps of immune-related DEGs were drawn using the pheatmap package of the R software. ClusterProfiler package was applied to perform Gene Ontology (GO) and Kyoto Encyclopedia of Genes and Genomes (KEGG) analyses of immune-related DEGs.

### Identification of Prognosis-Related DEGs

Log-rank Kaplan–Meier survival analysis was performed to seek out prognosis-related genes from aforementioned immune-related DEGs.

### Construction and Validation of the Prognostic Risk Model

One hundred HNSCC patients with clear p16 status and complete clinical information were used for identifying hub prognostic immune-related signature and constructing prognostic risk model. Twelve prognostic-related DEGs were analyzed by a stepwise multivariate Cox proportional hazards regression analysis. Six genes were extracted, and the risk score was established with the following formula: risk score = expression of gene 1× coefficient 1+ expression of gene 2× coefficient 2+ … expression of gene *n*× coefficient *n*. We calculated the area under the curve (AUC) by using the survivalROC package of R software to validate the predictive ability of the prognostic risk model. Patients were stratified into the high- and low-risk groups with a threshold of the median risk score, and Kaplan–Meier survival analysis was performed to estimate the survival difference. A heat map was also created. To further validate and develop the prognostic risk model, we explored it in all 479 HNSCC patients. Relationship between risk score and clinical characteristics in HNSCC patients was also investigated and visualized by ggplot2 package.

### Tumor-Infiltrating Immune Cells Fraction Calculation

We calculated tumor-infiltrating immune-cell fraction of each HNSCC patient by CIBERSORT algorithm (Newman et al., 2015). The reference 22 leukocyte expression data were downloaded from the website. Differences of immune-cell fraction between the high- and low-risk groups were visualized by pheatmap package and vioplot package. Single sample Gene Set Enrichment Analysis (GSEA) was applied to validate the differences of immune-cell fraction by GSVA and GSEAbase packages.

### Estimation of the Immunoreactivity

As hub immune response biomarkers, the expressions of PD-1, PD-L1, PD-L2, CTLA4, CD4, CD8A, and CD8B between the high- and low-risk groups were analyzed using Wilcoxon test. Based on machine learning and tumor genotype, immunophenoscore (IPS), is calculated and normalized with a range of 0 to 10. Higher scores are correlated with higher immunoreactivity (Charoentong et al., 2017). IPS was downloaded from The Cancer Immunome Atlas^4^.

### Statistical Analysis

A χ^2^ test or Fisher exact test was used for calculating the differences of clinical characteristics. Wilcoxon rank sum test was used to estimate the statistical significance of continuous variables. For constructing the prognostic risk model, univariate Cox regression analysis and multivariate Cox regression analysis were used. Survival analysis was performed by survival package (Holleczek and Brenner, 2013). All the aforementioned statistics were analyzed by R software (version 3.6.3). A two-sided *P* < 0.05 was considered to be statistically significant.

## Results

### Clinical Characteristics of HNSCC Patients

**FIGURE 1:**
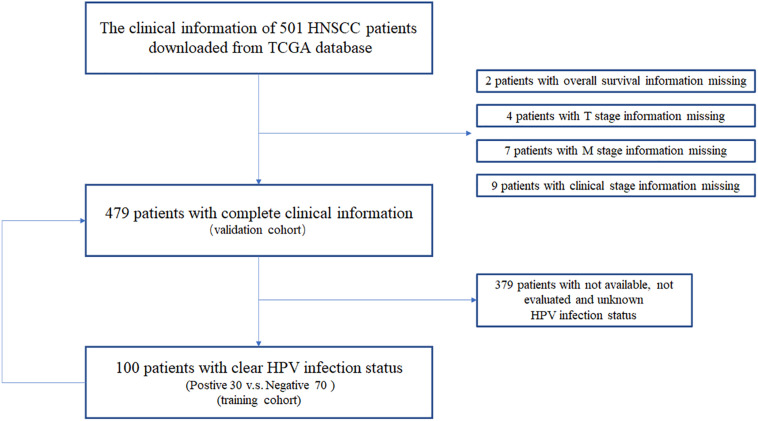
The clinical information acquisition and preprocessing of HNSCC patients from TCGA database.

We downloaded the clinical information of 479 HNSCC patients from TCGA data portal. In this study, we compared the results of HPV status identified by ISH or p16 testing, and there were few discrepancies (Supplementary Figure 1 and Supplementary Table 1). Thus, we chose p16 status, a widely used marker in clinic, to identify HPV-driven HNSCC tumor. We identified 30 p16-positive and 70 p16-negative HNSCC patients (Figure 1 and Table 1). Compared with p16-negative patients, p16-positive patients had more early T-stage diseases and fewer death events, albeit the latter did not meet a statistical difference.

**TABLE 1:** Clinical characteristics between p16-positive and p16-negative HNSCC patients.

Variables	Total (*n* = 100) *n*(%)	HPV positive (*n* = 30) *n*(%)	HPV negative (*n* = 70) *n*(%)	*P*-value^†^
**Age (years)**				0.0586
≤65	76(76.0%)	27(90.0%)	49(70.0%)	
>65	24(24.0%)	3(10.0%)	21(30.0%)	
**Gender**				0.2210
°Female		3(10%)	16(22.9%)	
°Male		27(90%)	54(77.1%)	
**Outcome**				0.0581
°Alive	72(72.0%)	26(86.7%)	46(65.7%)	
Dead	28(28.0%)	4(13.3%)	24(34.2%)	
**T stage**				0.0026**
T1	9(9.0%)	4(13.3%)	5(7.1%)	
T2	30(30.0%)	16(53.3%)	14(20.0%)	
T3	23(23.0%)	4(13.3%)	19(27.2%)	
T4	38(38.0%)	6(20.0%)	32(45.7%)	
**N stage**				0.1495
N0	32(32.0%)	7(23.3%)	25(35.7%)	
N1	15(15.0%)	2(6.7%)	13(18.6%)	
N2	49(49.0%)	20(66.7%)	29(41.4%)	
N3	1(1.0%)	0	1(1.4%)	
NX	3(3.0%)	1(3.3%)	2(2.9%)	
**M stage**				0.0251**
M0	97(97.0%)	27(90.0%)	70(100.0%)	
MX	3(3.0%)	3(10%)	0	
**Clinical stage**				0.8046
I	6(6.0%)	2(6.6%)	4(5.7%)	
II	14(14.0%)	5(16.7%)	9(12.9%)	
III	15(15.0%)	3(10.0%)	12(17.1%)	
IV	65(65.0%)	20(66.7%)	45(64.3%)	

*NX, unknown N stage; MX, unknown M stage. **^†^**By χ^2^ test or Fisher exact test where appropriate.*

### Identification of DEGs and Immune-Related DEGs

**FIGURE 2:**
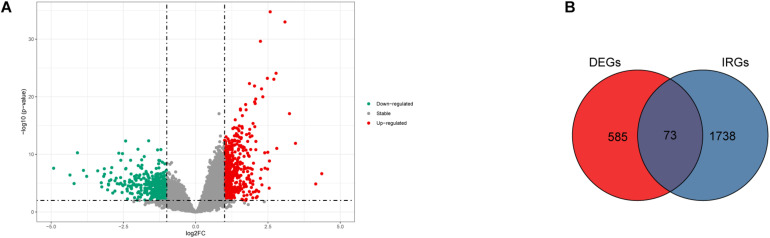
The DEGs and immune-related DEGs identified. **(A)** Volcano plot showing the differentially expressed genes in HNSCC positive and negative patients. **(B)** Venn plot showing the intersection of DEGs and immune-related genes.

**FIGURE 3:**
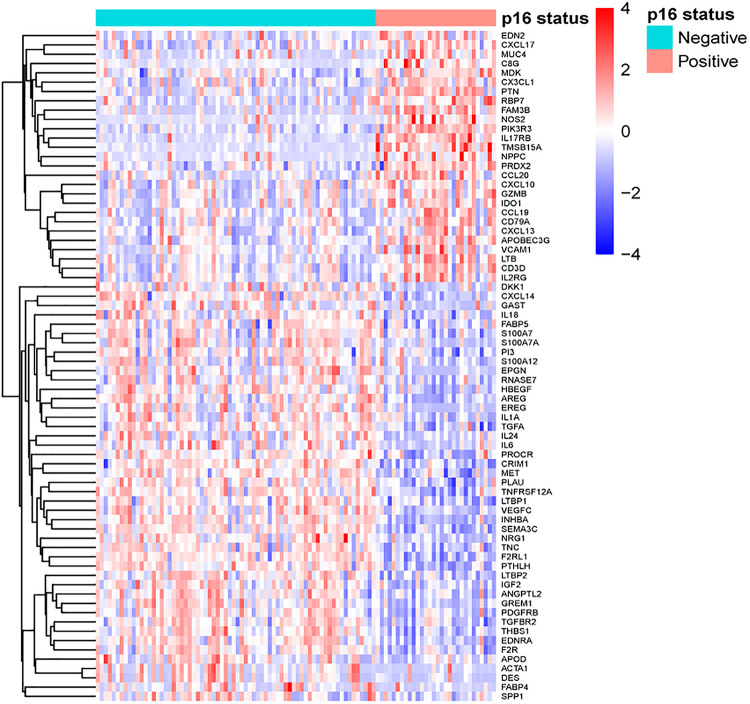
Heat map of 73 immune-related DEGs between HNSCC positive and negative patients.

**FIGURE 4:**
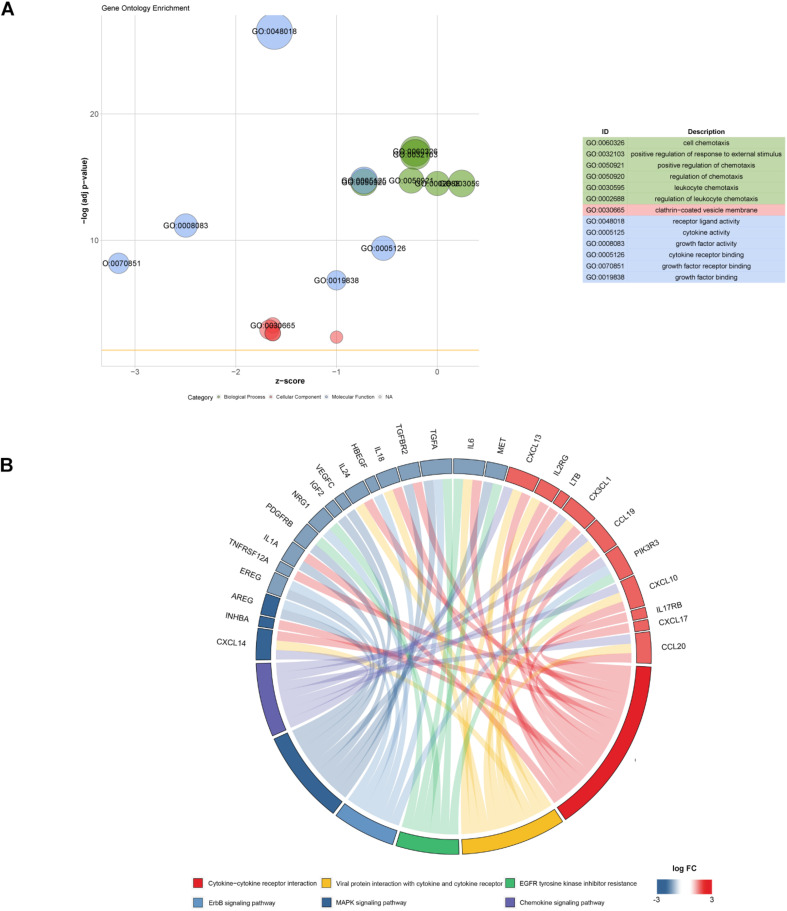
Function and pathway enrichment analysis of 73 immune-related DEGs. **(A)** Top significant GO enrichment analysis. **(B)** Six most significant KEGG pathways revealing that genes are involved in lots of immune-related pathways.

Gene expression profiles of p16-positive and p16-negative patients were obtained from the TCGA database. With a filter criterion of adjusted *P* < 0.05 and |log2 (fold change)| >1, 658 DEGs were identified by limma package (Figure 2A), of which 305 genes were up-regulated and 353 genes were down-regulated. Seventy-three immune-related DEGs were extracted from the intersection of DEGs and IRGs downloaded from ImmPort database (Figure 2B), containing 28 up-regulated genes and 45 down-regulated genes (Figure 3). GO and KEGG enrichment analyses were conducted to uncover the allocated biological process, cellular component, molecular function, and pathway of immune-related DEGs. The identified immune-related DEGs were found to be chiefly enriched in cytokine and receptor related functions and pathways. As shown in Figure 4A, the most significantly enriched terms were “cell chemotaxis,” “clathrin-coated vesicle membrane,” and “receptor ligand activity” under GO analysis. Top six enriched pathways were “cytokine–cytokine receptor interaction,” “Viral protein interaction with cytokine and cytokine receptor,” “EGFR tyrosine kinase inhibitor resistance,” “ErbB signaling pathway,” “MAPK signaling pathway,” and “chemokine signaling pathway” under KEGG analysis (Figure 4B).

### Identification of Prognosis-Related DEGs

**FIGURE 5:**
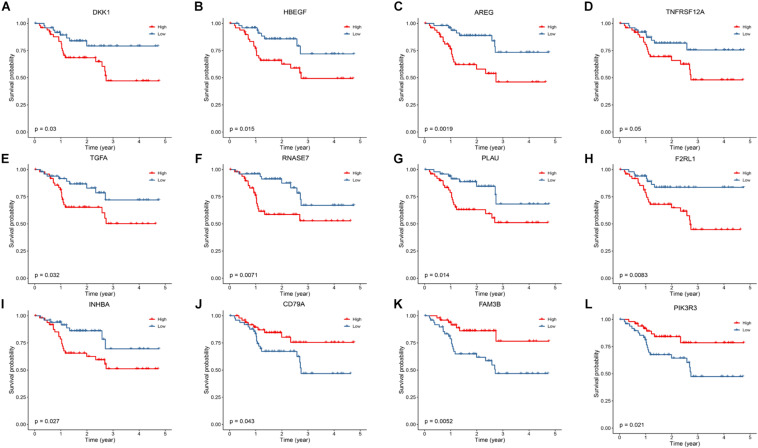
Kaplan–Meier analysis to identify prognosis-related DEGs. **(A–I)** The survival plot showed that expression of DKK1, HBEGF, AREG, TNFRSF12A, TGFA, RNASE7, PLAU, F2RL1 and INHBA are correlated with a favorable prognosis. **(J–L)** Low expression of CD79A, FAM3B and PIK3R3 are correlated with a worse prognosis.

Kaplan–Meier survival analysis was conducted to identify genes highly associated with OS of HNSCC patients. As shown in Figure 5, low expressions of DKK1, HBEGF, AREG, TNFRSF12, TGFA, RNASE7, PLAU, F2RL1, and IHNBA were significantly related with a favorable prognosis, whereas low expressions of CD79A, FAM3B, and PIK3R3 were linked to an ominous clinical outcome.

### Construction of Immune-Related Risk Model

**FIGURE 6:**
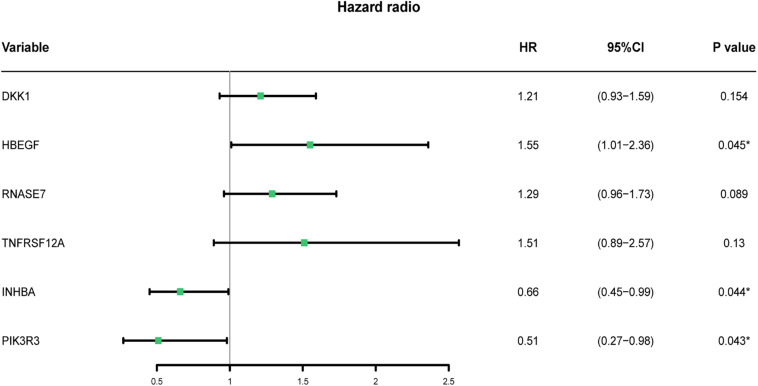
Risk model based on multivariate Cox analysis.

To explore the predictive value of aforementioned prognostic genes, we conducted a stepwise multivariate Cox regression analysis. Six of the 12 genes were extracted and considered significantly linked to the OS of 100 HNSCC patients. Forest plot showed DKK1, HBEGF, RNASE7, and TNFRSF12A were associated with poor outcomes, and INHBA and PIK3R3 were associated with favorable outcomes (Figure 6 and Table 2). Based on the multivariate Cox regression analysis, we estimated the values of six genes and constructed the risk scores as the following formula: risk score = (0.194662 × expression of DKK1) + (0.435235 × expression of HBEGF) + (0.254406 × expression of RNASE7) + (0.411604 × expression of TNFRSF12A) + (−0.41064 × expression of INHBA) + (−0.67105 × expression of PIK3R3).

**TABLE 2:** Characteristic and coefficients of risk model genes.

**Gene**	**Log FC**	**Regulation**	**Coefficient**	**HR (95% CI)**	***P*-value**
DKK1	–1.089	Down	0.194662	1.21 (0.93–1.59)	0.154
HBEGF	–1.159	Down	0.435235	1.54 (1.01–2.36)	0.045*
RNASE7	–1.378	Down	0.254406	1.28 (0.96–1.73)	0.089
TNFRSF12A	–1.343	Down	0.411604	1.51 (0.89–2.57)	0.130
INHBA	–2.347	Down	−0.41064	0.66 (0.45–0.99)	0.044*
PIK3R3	1.255	Up	−0.67105	0.51 **(0.27–0.98)**	0.043*

CI, confidence interval; HR, hazard radio; FC, fold change. *p < 0.05, **p < 0.01, ***p < 0.001.

**FIGURE 7:**
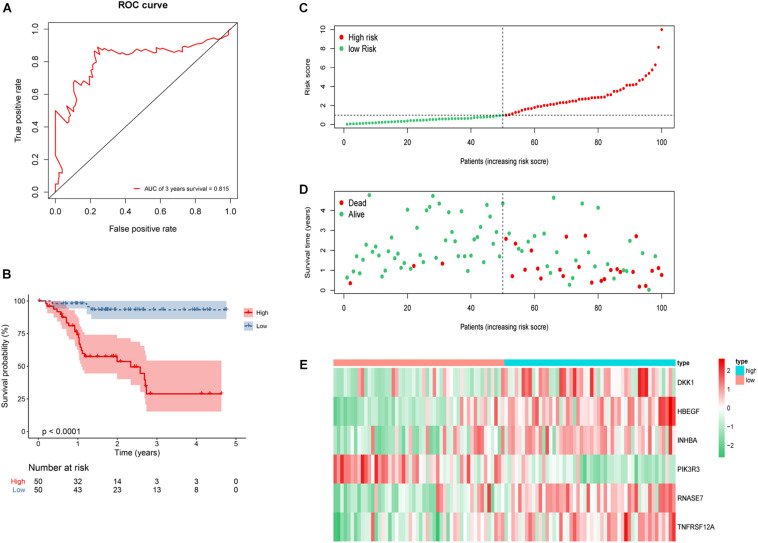
Construction of six-gene risk model in 100 HNSCC patients. **(A)** Receiver operating characteristic (ROC) curve of 3 years overall survival (OS). The area under the red ROC curve is 0.815. **(B)** Kaplan–Meier survival curve of the OS in the high- and low-risk groups. **(C)** The distribution plot of risk score. **(D)** The survival status plot associated with risk score. **(E)** Six hub DEGs’ expression heat map of the high- and low-risk groups.

According to the formula, we calculated the risk score of every HNSCC patient. We then divided the patients into low-risk group (*n* = 50) and high-risk group (*n* = 50) by the median risk score. A receiver operating characteristic curve of 3-year OS (AUC = 0.815) was drawn to estimate the predictive ability of the risk model (Figure 7A). High-risk score group revealed a worse clinical outcome compared with low-risk group by Kaplan–Meier analysis (Figure 7B). The distributions of risk score and patients’ survival status are displayed in Figures 7C,D. Heat map revealed gene expression differences between the high- and low-risk groups (Figure 7E).

### Validation of the Risk Model in Total HNSCC Patients

**FIGURE 8:**
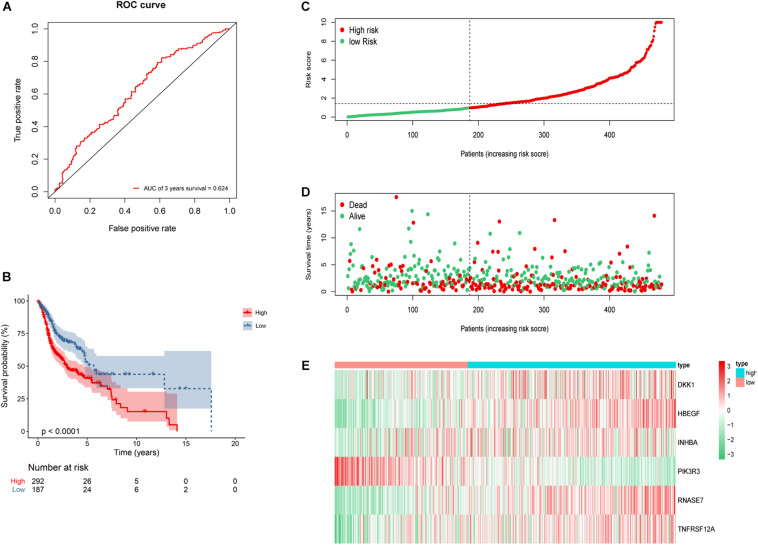
Construction of 6 genes risk model in whole 479 HNSCC patients. **(A)** ROC curve of 3 years overall survival (OS). The area under the red ROC curve is 0.624. **(B)** Kaplan–Meier survival curve of the OS in the high- and low-risk groups. **(C)** The distribution plot of risk score. **(D)** The survival status plot associated with risk score. **(E)** 6 hub DEGs expression heat map of the high- and low-risk groups.

To further validate the reliability of the constructed risk model, the risk score of whole 479 HNSCC patients were calculated and divided into either low-risk group (*n* = 187) or high-risk group (*n* = 292). The AUC of 3-year OS was 0.624 (Figure 8A). Kaplan–Meier survival curves revealed statistically significant difference between the high- and low-risk groups (*P* < 0.001), and the high-risk group was associated with a worse clinical outcome (Figure 8B). Based on the risk score, the distribution, survival status, and gene expression differences were also evaluated and visualized (Figures 8C–E). Similar results were observed in whole 479 TCGA HNSCC patients, illustrating the reliable predictive ability of risk model.

### Association Between the Risk Score and Clinical Characteristics and Their Prognostic Roles

**FIGURE 9:**
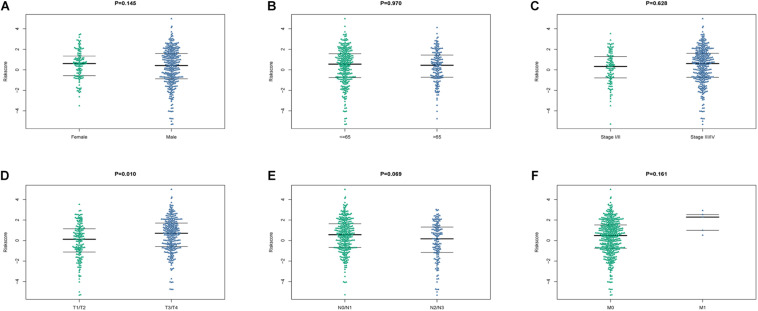
The relationship between the risk score and clinical characteristics. **(A–F)** Beeswaram plot showed the distributions and differences between male and female **(A)**, age >65 years old and ≤ 65 years old **(B)**, early stage and advanced stage **(C)**, T stage **(D)**, N stage **(E)** and M stage **(F)**.

The relationship between the risk model and clinical characteristics were analyzed by univariate and multivariate Cox regression analyses. Age older than 65 years, advanced N stage, and metastatic disease were significantly associated with unfavorable prognosis (Table 3). Risk score could serve as an independent predictive factor for HNSCC patients. Relationship between risk score and clinical characteristics were also analyzed (Figure 9), in which risk score and T stage seemed closely linked to each other.

**TABLE 3:** Univariate analysis and multivariate analysis of risk model.

**Variables**	**Univariate analysis**		**Multivariate analysis**	
	**HR (95% CI)**	***P*-value**	**HR (95% CI)**	***P*-value**
Age	1.40	0.018*	1.39	0.025*
	(1.05–1.85)		(1.04–1.86)	
Sex	0.74	0.051	0.82	0.212
	(0.55–1.00)		(0.66–1.11)	
T stage	1.08	0.256	1.15	0.278
	(0.94–1.25)		(0.88–1.50)	
N stage	1.13	0.104	1.26	0.034*
	(0.97–1.32)		(1.01–1.58)	
M stage	4.70	0.002**	4.82	0.004**
	(1.73–12.78)		(1.64–14.14)	
Clinical stage	1.09 (0.94–1.28)	0.237	0.84	0.315
			(0.60–1.17)	
Risk score	1.06	0.001**	1.06	0.003**
	(1.02–1.10)		(1.01–1.10)	

CI, confidence interval; HR, hazard radio. *p < 0.05, **p < 0.01, ***p < 0.001.

### Differential Immune Landscape in the High- and Low-Risk Groups

**FIGURE 10:**
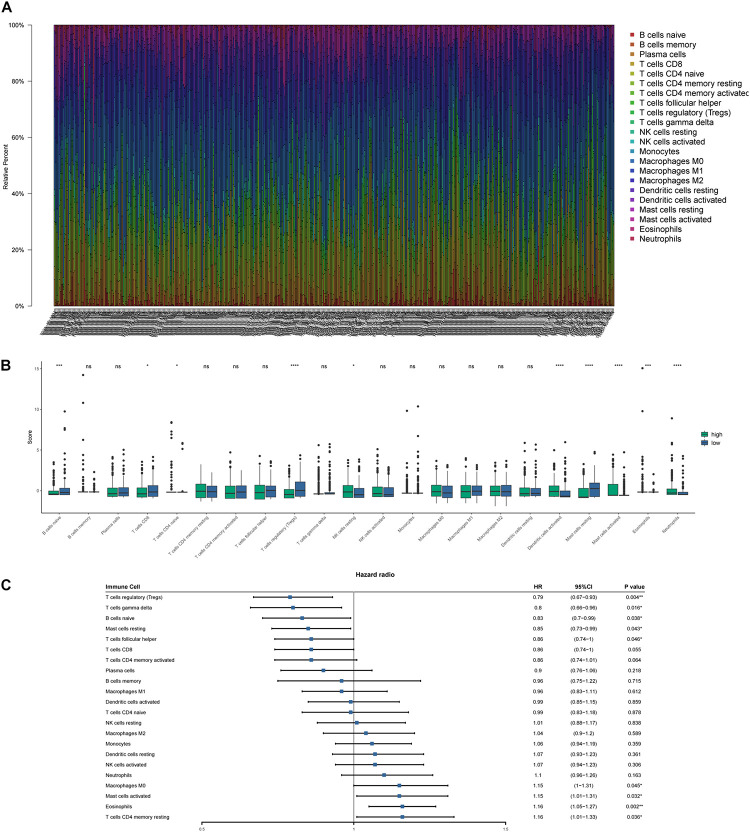
Tumor-infiltrating immune-cell fraction estimated by CIBERSORT. **(A)** The proportion of immune cells in every sample. **(B)** The comparison of immune-cell fractions between the high- and low-risk groups. **(C)** Forest plot based on univariate Cox analysis. *p < 0.05, **p < 0.01, ***p < 0.001, ****p < 0.0001.

**FIGURE 11:**
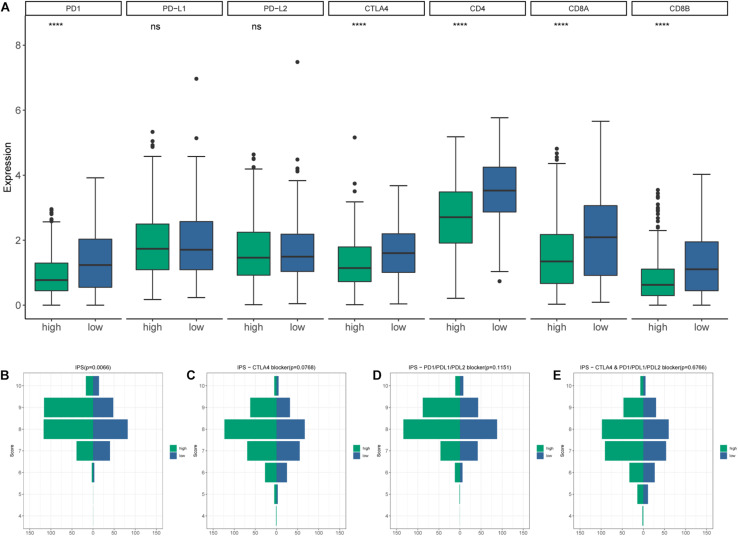
**(A)** The differential expression of immune checkpoint inhibitor (ICI)–associated biomarkers. **(B)** IPS score distribution plot. **(C)** IPS–CTLA4 blocker score distribution plot. **(D)** IPS–PD1/PDL1/PDL2 blocker score distribution plot. **(E)** IPS–CTLA4 and PD1/PDL1/PDL2 blocker score distribution plot.

CIBERSORT algorithm was applied to demonstrate the relationship between risk score and tumor-infiltrating immune-cell fractions. Samples with *P* < 0.05 were considered statistically different. Immune-cell fraction of 448 of 479 HNSCC patients was calculated for further analyses (Figure 10A). The abundance of immune cells between 268 high-risk and 180 low-risk patients were normalized and compared by Wilcoxon rank sum test (Figure 10B). CD8^+^ T cells, Tregs, naive B cells, naive CD4^+^ T cells, and resting mast cells were evidently abundant in low-risk TME, whereas fewer resting NK cells, activated dendritic cells, activated mast cells, eosinophils, and neutrophils were present. A univariate Cox regression analysis–based forest plot displayed the association between immune cells and OS of HNSCC patients (Figure 10C). Using ssGSEA analysis, we validated the differences of immune cells between the low- and high-risk groups, and similar results were observed (Supplementary Figure 2). We further investigated the expression of hub biomarkers of ICI response (Figure 11A). The expressions of *PD-1*, *CTLA4*, *PD-L2*, *CD4*, *CD8A*, and *CD8B* were significantly higher in low-risk samples. Differences between two groups demonstrated that the specific changes of tumor-infiltrating immune cells might be associated with the OS of HNSCC patients. The scores of IPS and IPS with CTLA4 blockers, IPS with PD1/PDL1/PDL2 blockers, IPS with CTLA4, and PD1/PDL1/PDL2 blockers were calculated (Figures 11B–E), and IPS score in low-risk group was significantly higher compared with that in the high-risk group.

## Discussion

The incidence of HNSCC is continuously increasing around the world and may surpass that of cervical cancer (Marur and Forastiere, 2016). However, only a small proportion of HNSCC patients respond to and gain benefits from targeted drugs and ICI therapies (Concha-Benavente et al., 2016; Ferris et al., 2018). It is urgent and critical to find inventive ways to predict response to ICI treatment. Accumulating evidences have documented the potential effects of HPV on HNSCC patients in several aspects including genomic integration, carcinogenesis, tumor angiogenesis, and TME (Troy et al., 2013; Parfenov et al., 2014; Cao et al., 2019; Wookey et al., 2019). Therefore, HPV status of HNSCC patients provides a promising entry point for predicting response to ICI therapy and unearthing the elusive molecular mechanism. Within this study, we aim to further inspect how HPV infection impacts malignant gene expression profile and response of HNSCC to ICI therapy. In addition, it is of vital importance to develop a prognostic immune signature based on immune-related biomarkers, which might enhance the therapeutic efficacy of various HNSCC patients.

It has been demonstrated that the p16 protein is overexpressed in the majority (82.2%) of HPV-associated (defined as HPV DNA positive) oropharyngeal carcinoma, and the detection of p16 by immunohistochemical staining is routinely used as a reliable surrogate marker in clinical practice and research (Li et al., 2004; El-Naggar and Westra, 2012; Ndiaye et al., 2014). Compared with other detection methods such as HPV DNA and E6/E7 mRNA detection by ISH that are usually expensive and time-consuming, p16 immunohistochemical staining is relatively inexpensive, timesaving, and convenient, so it is widely used for clinical detection and recommended by the modified eighth AJCC/UICC cancer staging system regarding HPV positivity (Lydiatt et al., 2017).

Previous studies pointed out that HPV infection and TME seem impossibly remote. HPV-positive status is a proficient effector in lymphocyte recruitment in the TME of cervical cancer (Wood et al., 2016). Along similar lines, in head and neck cancer, HPV-positive cell lines are competent in lymphocyte recruitment and cytokine secretion compared to HPV-negative cell lines (Stone et al., 2014). Moreover, HPV-positive tumor could polarize macrophages toward classically activated phenotype (M1) and augment antitumoral IL-6 secretion (Chen et al., 2019). In our study, it is highly likely that immune-related DEGs were allocated to cytokines and receptors–related functions and pathways, indicating that HPV infection might serve a dominant role in shaping the TME by altering the expression and cell recognition of cytokines, with the molecular mechanisms remaining uncertain.

Because of the complexity and heterogeneity of tumor biological behavior and immune response, it is unreliable to apply one single biomarker to fully illustrate and predict the prognosis and therapeutic response. Therefore, prediction model based on multibiomarkers may be a more effective and accurate tool, stratifying HNSCC patients into high- and low-risk subgroups and identifying those that might achieve clinical benefits from ICIs or other immunotherapies.

We developed and validated an immune-related prognostic risk model that gave an accurate prediction of survival outcome for HNSCC patients based on immune-related DEGs. Most of these genes hold a decisive place in expression of cytokine and its receptors. Overexpression of DKK1, an identified proinflammatory cytokine in quite a number of cancers (Chae and Bothwell, 2019), could boost malignant cell proliferation, migration, and invasiveness and give rise to unfavorable clinical outcomes in HNSCC (Shi et al., 2014; Gao et al., 2018). HBEGF serves as a crucial component of EGFR, mediating tumor cell proliferation and bringing about cetuximab resistance (Hatakeyama et al., 2010; Huang et al., 2014). Therefore, HBEGF expression contributes to locating the potential beneficiaries of cetuximab treatment. INHBA encodes proteins of TGF-β superfamily members, which is considered as hub gene in lymphatic metastasis and predictor of unsatisfying OS in HNSCC patients (Kelner et al., 2015; Chang et al., 2016). Moreover, in terms of PIK3R3, RNASE7, and TNFRSF12A, similar results have been obtained in other researches, indicating that these genes might be engaged in immune-related pathways and promote tumor progression (Scola et al., 2012; Wang et al., 2015; Eichler et al., 2016; Yang et al., 2018; Sun and Feng, 2020). In this study, expression patterns of these genes in HNSCC patients were suggestive to be capable of modifying the TME, adjusting immune response, initiating tumorigenesis, and predicting prognosis. Given the high AUC and statistically significant differences of OS between two groups, immune-related risk model functioned as a strong indicator of OS in HNSCC patients. Furthermore, the risk score, correlated with T stage, might act as an independent prognostic factor.

Composition of tumor-infiltrating immune cells was estimated by CIBERSORT and ssGSEA for each sample. Abundance of CD8^+^ T cells and Tregs, was richer in the low-risk group and was correlated with favorable prognosis. In this study, patients with higher CD8^+^ T cell infiltration had a better clinical outcome, which was consistent with previous investigations (Balermpas et al., 2014; de Ruiter et al., 2017). It is a well-acknowledged fact that Tregs work as an adverse factor in antitumor immunity. Quite the reverse, recent studies demonstrated that compared with HPV-negative HNSCC patients, HPV-positive patients have a higher level of Treg infiltration (Mandal et al., 2016) and usually a longer survival time (Mirghani et al., 2019). Likewise, higher infiltration of Tregs and expression of immune checkpoints are revealed in HNSCC microenvironment, indicating a complicated relationship between Tregs and the antitumor immune response (Mandal et al., 2016; Saloura et al., 2019). High-degree infiltration of Tregs and its correlation with better prognosis observed in this study were possibly due to the interaction between Tregs and other immune components in the TME.

The hub biomarkers of ICI were explored. Low-risk patients displayed up-regulation of PD-1 and CTLA4. Meanwhile, in the risk model, patients in the low-risk group had a remarkably increased IPS score. Aleix and colleagues described that PD-1 gene expression is associated with longer progression-free survival (Prat et al., 2017). Furthermore, anti-CTLA4 therapy could enhance antitumor effect via Treg exhaustion (Selby et al., 2013; Simpson et al., 2013). Hence, high infiltration of Tregs in the low-risk group may be conducive to combination of ICIs and depletion strategies targeting Tregs. Collectively, these results demonstrated that the TME of low-risk group had a higher immunoreactivity. The risk model might hold the capacity to determine tumor response to immunotherapy in HNSCC patients. IPS with CTLA4 and/or PD1/PDL1/PDL2 blocker scores were of no statistical significance, revealing that the prediction value of the risk model in response to immunotherapy was not fully understood and required further research.

In conclusion, we constructed a gene model based on HPV status, providing a possible method to estimate the TME and predict OS and response to immunotherapy in HNSCC patients. Despite the aforementioned promising results, there still exist some limitations. First, the datasets are based on a public database. Second, the number of HPV-positive HNSCC patients is relatively small. Whether a larger amount of cases enrolled would affect the study results remains unknown. In addition, we focused on the DEGs within p16-positive and -negative groups, which is only one of the potentially useful stratifying strategies in head and neck cancers. Further, we focused only on the biological function and pathway enrichment of immune-related DEGs due to limited research scope, and many potential DEGs failed to be investigated. However, the promising results carry more weight over these limitations.

## Conclusion

This study has examined the differential expression pattern between the HPV-positive group and HPV-negative group defined by p16 status, and 12 DEGs were perceived as prognosis-related genes. A six-immune-related DEGs–based risk model was constructed, and the predictive ability, TME, and ICI response were consequently estimated. The risk model is reliable and provides a possible method for clinical outcome prediction and immunotherapy improvement.

## Data Availability Statement

Publicly available datasets were analyzed in this study. This data can be found here: https://portal.gdc.cancer.gov/.

## Ethics Statement

Ethical review and approval was not required for the study on human participants in accordance with the local legislation and institutional requirements. Written informed consent for participation was not required for this study in accordance with the national legislation and the institutional requirements.

## Author Contributions

XL: substantial contributions to conception, design, and data analysis. JC: bioinformatics algorithms and analysis instruction. WL: datasets acquisition and data preprocessing. ZZ and JL: construction of stepwise multivariate Cox analysis and validation. YPG, XJ, and QW: drafting the article. YG and QW: supervision of the study and critical revision of manuscript. CX: conception of the study, and design the workflow. All authors contributed to manuscript revision, read and approved the submitted version.

## Conflict of Interest

The authors declare that the research was conducted in the absence of any commercial or financial relationships that could be construed as a potential conflict of interest.

## Footnotes

1 https://portal.gdc.cancer.gov/

2 https://immport.niaid.nih.gov

3 https://www.r-project.org/

4 https://tcia.at/home
