# Supplementary material for: Systematic Profiling of Immune Risk Model to Predict Survival and Immunotherapy Response in Head and Neck Squamous Cell Carcinoma
Source: Front Genet. 2020 Oct 16;11:576566. doi: 10.3389/fgene.2020.576566 (PMC7596453; doi:10.3389/fgene.2020.576566)
Supplement: Supplementary file 1 [file Table_1.DOCX]

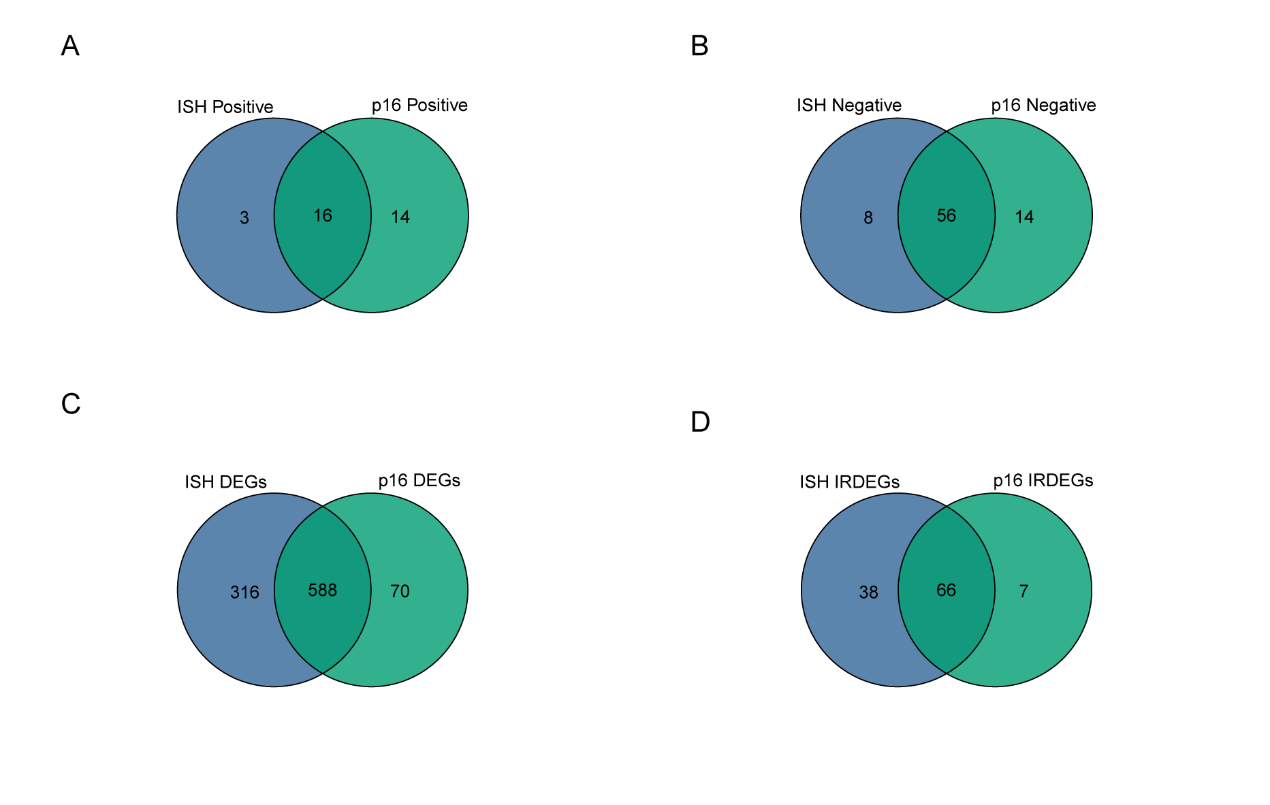
**Supplementary Figure 1** **Venn plots displayed overlaps and differences of immune-related differential expressed genes (IRDEGs) based on *in situ* hybridization (ISH) or p16 immunoshitochemical staining methods.** **A-B**. HPV status of patients defined by ISH or p16 immunoshitochemical staining methods. **C-D.** The DEGs and IRDEGs between HPV- positive and negative groups based on ISH or p16 immunoshitochemical staining methods. IRDEGs: immune-related differential expressed genes; ISH: *in situ* hybridization.


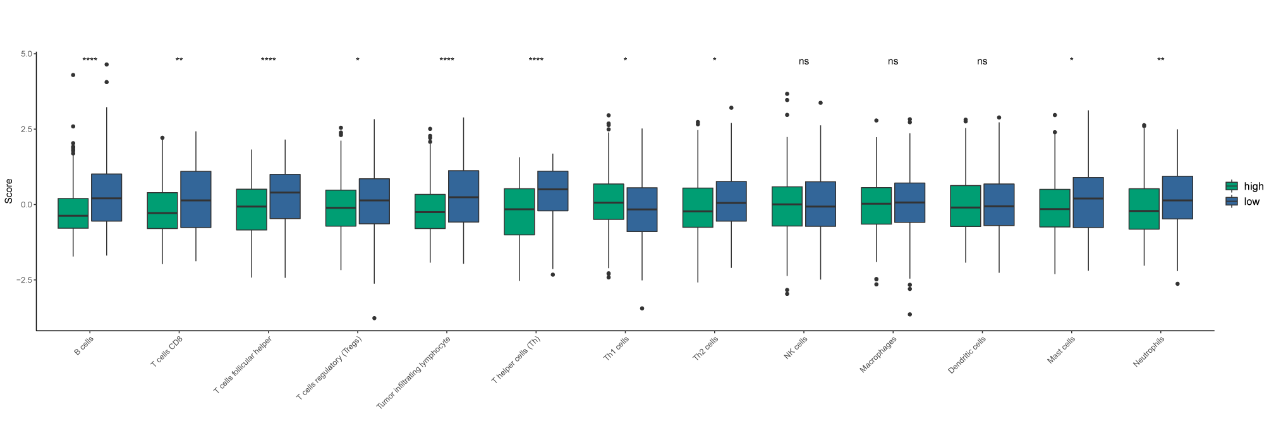


**Supplementary Figure 2 Tumor-infiltrating immune cells fraction estimated by ssGSEA.** * p < 0.05, ** p < 0.01, *** p < 0.001, **** p < 0.0001

**Supplementary Table** immune related DEGs between HPV positive and HPV negative identified by ISH method.

| **gene** | **logFC** | **P.Value** | **regulated** |
| --- | --- | --- | --- |
| PIK3R3 | 1.604464 | 3.94E-18 | UpRegulated |
| TMSB15A | 2.080228 | 1.37E-13 | UpRegulated |
| BRD8 | 1.066309 | 1.90E-13 | UpRegulated |
| IL17RB | 1.644534 | 2.06E-13 | UpRegulated |
| TYK2 | 1.096507 | 2.43E-13 | UpRegulated |
| TNFRSF13C | 1.128213 | 2.73E-12 | UpRegulated |
| SEMA6A | 1.226758 | 1.74E-11 | UpRegulated |
| FAM3B | 2.551265 | 2.59E-11 | UpRegulated |
| APOBEC3C | 1.101841 | 3.07E-10 | UpRegulated |
| PTN | 2.488233 | 1.24E-09 | UpRegulated |
| NOS2 | 2.271909 | 1.47E-09 | UpRegulated |
| APOBEC3G | 1.386807 | 5.92E-09 | UpRegulated |
| PRDX2 | 1.319936 | 3.17E-08 | UpRegulated |
| TNFRSF14 | 1.111854 | 3.34E-08 | UpRegulated |
| MDK | 1.826258 | 9.14E-08 | UpRegulated |
| ZAP70 | 1.018304 | 1.30E-07 | UpRegulated |
| CD19 | 1.024914 | 2.99E-07 | UpRegulated |
| CX3CL1 | 1.982642 | 3.89E-07 | UpRegulated |
| PDCD1 | 1.085702 | 5.91E-07 | UpRegulated |
| CD3D | 1.532136 | 1.49E-06 | UpRegulated |
| SOCS1 | 1.103967 | 1.55E-06 | UpRegulated |
| CD40 | 1.091422 | 1.62E-06 | UpRegulated |
| IDO1 | 2.291637 | 2.36E-06 | UpRegulated |
| CD8B | 1.148876 | 3.25E-06 | UpRegulated |
| GZMB | 1.530944 | 6.22E-06 | UpRegulated |
| NPPC | 1.360327 | 6.52E-06 | UpRegulated |
| CD8A | 1.469457 | 7.05E-06 | UpRegulated |
| MUC4 | 1.927199 | 8.13E-06 | UpRegulated |
| C8G | 1.213013 | 8.50E-06 | UpRegulated |
| RBP7 | 1.322983 | 1.61E-05 | UpRegulated |
| CD3E | 1.29549 | 2.23E-05 | UpRegulated |
| LTB | 1.211934 | 2.48E-05 | UpRegulated |
| IL2RG | 1.349025 | 2.85E-05 | UpRegulated |
| CD79B | 1.009574 | 5.18E-05 | UpRegulated |
| CD79A | 1.857127 | 5.55E-05 | UpRegulated |
| CXCL17 | 2.18215 | 5.57E-05 | UpRegulated |
| CCL20 | 2.064092 | 5.61E-05 | UpRegulated |
| IRF1 | 1.084846 | 7.49E-05 | UpRegulated |
| CXCR3 | 1.065165 | 9.36E-05 | UpRegulated |
| HLA-DMB | 1.034695 | 0.000323 | UpRegulated |
| PRF1 | 1.087036 | 0.000341 | UpRegulated |
| BMP7 | 1.159835 | 0.000423 | UpRegulated |
| VCAM1 | 1.267842 | 0.000508 | UpRegulated |
| WFDC2 | 2.096305 | 0.000994 | UpRegulated |
| CXCL10 | 1.974752 | 0.001064 | UpRegulated |
| CXCL13 | 1.230557 | 0.001866 | UpRegulated |
| PTGDS | 1.194814 | 0.001979 | UpRegulated |
| CCL19 | 1.602622 | 0.002129 | UpRegulated |
| CXCL9 | 1.632165 | 0.002881 | UpRegulated |
| HLA-DRB5 | 1.13168 | 0.003812 | UpRegulated |
| CD74 | 1.054691 | 0.004499 | UpRegulated |
| CCL5 | 1.106764 | 0.004962 | UpRegulated |
| DUOX2 | 1.041782 | 0.010606 | UpRegulated |
| F2RL1 | -1.9961 | 1.08E-13 | DownRegulated |
| INHBA | -2.65775 | 6.97E-13 | DownRegulated |
| SEMA3C | -1.9862 | 2.28E-10 | DownRegulated |
| CXCL14 | -3.15895 | 1.05E-09 | DownRegulated |
| THBS1 | -2.04776 | 2.32E-08 | DownRegulated |
| TNC | -2.05141 | 1.13E-07 | DownRegulated |
| CRIM1 | -1.13279 | 2.56E-07 | DownRegulated |
| EDNRA | -1.1542 | 2.80E-07 | DownRegulated |
| PLTP | -1.13502 | 3.17E-07 | DownRegulated |
| MET | -1.05 | 4.28E-07 | DownRegulated |
| PTHLH | -2.20242 | 4.93E-07 | DownRegulated |
| GREM1 | -1.48172 | 5.04E-07 | DownRegulated |
| HBEGF | -1.30742 | 1.05E-06 | DownRegulated |
| PDGFRB | -1.46069 | 1.59E-06 | DownRegulated |
| VEGFC | -1.43192 | 2.10E-06 | DownRegulated |
| RNASE7 | -1.96767 | 2.31E-06 | DownRegulated |
| AREG | -2.10857 | 2.50E-06 | DownRegulated |
| IGF2 | -1.49497 | 3.37E-06 | DownRegulated |
| ANGPTL2 | -1.37574 | 4.73E-06 | DownRegulated |
| IL18 | -1.24916 | 5.48E-06 | DownRegulated |
| INHBB | -1.19844 | 1.02E-05 | DownRegulated |
| NRG1 | -1.25819 | 1.09E-05 | DownRegulated |
| PLAU | -1.20161 | 1.27E-05 | DownRegulated |
| S100A7A | -2.37718 | 1.76E-05 | DownRegulated |
| TGFA | -1.08266 | 1.76E-05 | DownRegulated |
| TNFRSF12A | -1.07787 | 1.90E-05 | DownRegulated |
| PROCR | -1.11399 | 2.01E-05 | DownRegulated |
| CXCL5 | -1.40559 | 3.51E-05 | DownRegulated |
| F2R | -1.09706 | 3.52E-05 | DownRegulated |
| EPGN | -1.68756 | 3.79E-05 | DownRegulated |
| PI3 | -2.79281 | 4.39E-05 | DownRegulated |
| LTBP1 | -1.27865 | 4.46E-05 | DownRegulated |
| CLEC11A | -1.02438 | 4.73E-05 | DownRegulated |
| EREG | -1.70968 | 6.63E-05 | DownRegulated |
| IL24 | -1.57501 | 0.000117 | DownRegulated |
| GAST | -1.32642 | 0.000187 | DownRegulated |
| IL1A | -1.66376 | 0.000235 | DownRegulated |
| CCL24 | -1.02495 | 0.000555 | DownRegulated |
| PGLYRP4 | -1.11664 | 0.000563 | DownRegulated |
| IL1B | -1.20262 | 0.000615 | DownRegulated |
| IL6 | -1.28392 | 0.000621 | DownRegulated |
| CSF2 | -1.19928 | 0.001013 | DownRegulated |
| S100A7 | -2.35833 | 0.001758 | DownRegulated |
| S100A12 | -1.57364 | 0.002268 | DownRegulated |
| SLURP1 | -2.02092 | 0.003123 | DownRegulated |
| DES | -2.14027 | 0.006484 | DownRegulated |
| AZGP1 | -1.06605 | 0.006604 | DownRegulated |
| CHGB | -1.00285 | 0.007398 | DownRegulated |
| CRABP2 | -1.0951 | 0.010337 | DownRegulated |
| FABP5 | -1.25936 | 0.013687 | DownRegulated |
| FABP4 | -1.27571 | 0.017635 | DownRegulated |
